# Aortic Arch Calcification Predicts Patency Loss of Arteriovenous Fistula in End-Stage Renal Disease Patients

**DOI:** 10.1038/srep24943

**Published:** 2016-04-22

**Authors:** Yit-Sheung Yap, Kai-Ting Ting, Wen-Che Chi, Cheng-Hao Lin, Yi-Chun Liu, Wan-Long Chuang

**Affiliations:** 1 Graduate Institute of Clinical Medicine, College of Medicine, Kaohsiung Medical University, Kaohsiung, Taiwan; 2 Division of Nephrology, Department of Internal Medicine, Yuan’s General Hospital, Kaohsiung, Taiwan; 3 Division of Gastroenterology, Department of Internal Mednicine, Yuan’s General Hospital, Kaohsiung, Taiwan; 4 Division of Hepatobiliary, Department of Internal Medicine, Kaohsiung Medical University Hospital, Kaohsiung Medical University, Kaohsiung, Taiwan

## Abstract

Aortic arch calcification (AAC) is recognized as an important cardiovascular risk factor in patients with end-stage renal disease (ESRD). The aim of the study was to evaluate the impact of AAC grade on patency rates of arteriovenous fistula (AVF) in this specific population. The data of 286 ESRD patients who had an initial AVF placed were reviewed. The extent of AAC identified on chest radiography was divided into four grades (0–3). The association between AAC grade, other clinical factors, and primary patency of AVF was then analyzed by Cox proportional hazard analysis. The multivariate analysis demonstrated that the presence of AAC grade 2 (hazard ratio (95% confidence interval): 1.80 (1.15–2.84); p = 0.011) and grade 3 (3.03 (1.88–4.91); p < 0.001), and higher level of intact-parathyroid hormone (p = 0.047) were associated with primary patency loss of AVF. In subgroup analysis, which included AVF created by a surgeon assisted with preoperative vascular mapping, only AAC grade 3 (2.41 (1.45–4.00); p = 0.001), and higher intact-parathyroid hormone (p = 0.025) level were correlated with AVF patency loss. In conclusion, higher AAC grade and intact-parathyroid hormone level predicted primary patency loss of AVF in an ESRD population.

Vascular access dysfunction, particularly loss of primary functional patency of a surgically created access, is a major contributor to the hospitalization of hemodialysis patients and their overall morbidity and mortality^1^. According to 2014 United States Renal Data System (USRDS) report, more than 80% of end-stage renal disease (ESRD) patients worldwide including Taiwan choose hemodialysis as their first-line renal replacement therapy^2^,^3^. Thus, a reliable vascular access is critical for delivery of adequate hemodialysis and maintaining good quality of life in these populations.

Vascular calcification as well as aortic arch calcification (AAC) is highly prevalent in ESRD patients^4^. Chronic inflammation, hyperphosphatemia, and an increased calcium–phosphate product and deficiencies of calcification inhibitors are potential factors that contribute to progressive vascular calcification^5^,^6^. Recently, several reports have demonstrated that AAC is highly correlated with calcification of coronary artery, heart valve or extra- and intracranial carotid artery, reflecting an underlying systemic vascular atherosclerotic process^7^,^8^. Moreover, it is also an independent predictor of all-cause and cardiovascular mortality in ESRD patients^9^. Therefore, AAC could be the marker of systemic vascular calcification and important cardiovascular risk factor for patients on dialysis.

There also appears to be a positive correlation between AAC and arterial micro-calcification of vascular access^10^. Interestingly, preexisting radial artery macro-calcifications could predict the poor patency rate of radiocephalic fistulas in diabetic hemodialysis patients^11^. It is proposed that calcified vessels may increase arterial stiffness, which limits arterial dilation for adequate blood flow, and then contributes to thrombosis and access failure^12^. Otherwise, a prior study also established that patients with low fetuin A level, indicated as being more prone to vascular calcification, were associated with a higher risk of patency loss of vascular access in either native arteriovenous fistula (AVF) or arteriovenous graft (AVG)^13^. Taken together, vascular access calcification is an independent risk factor for poor access outcome, but clinical significance of AAC on patency rate is still unclear in these ESRD patients.

Although electron beam tomography (EBCT) or multidetector computed tomography (MDCT) are reliable in detecting aortic calcification, these imaging modalities are not routinely used^14^,^15^. In contrast, chest radiography is a non-invasive and inexpensive tool for the identification of aortic arch calcification. In addition, compared with plain radiography hand film, chest radiography is easier and more precise for calcification grading. Thus, the main objectives of this study were firstly to investigate the prevalence of different grades of AAC, and secondly, to study whether AAC predicted primary patency loss of AVF in ESRD patients by using simple, routine chest radiography.

## Results

### Comparison of clinical characteristics according to the presence of higher AAC grade

Mean age of the study subjects was 62.5 ± 13.1 years and the number of males was 163 (57.0%). The proportion of coronary artery disease (CAD), cerebrovascular disease (CVA), peripheral artery disease (PAD), diabetes and hypertension were 31.8%, 18.2%, 10.1%, 74.8% and 97.6% respectively. Among these ESRD patients, 60 patients were categorized as Grade 0 (21.0%), 72 as Grade 1 (25.2%), 90 as Grade 2 (31.5%) and 64 as Grade 3 (22.4%). Table 1 demonstrates the comparison of clinical findings between lower and higher AAC grades. The patients with a higher AAC grade were older (p < 0.001) and comprised a higher proportion of the female gender (p = 0.036), than patients with lower AAC grade. Besides, they had higher incidence of CAD (p < 0.001), CVA (p = 0.002) and diabetes (p = 0.008). Of note, lower blood pressure levels including systolic blood pressure (SBP) (p = 0.046) and diastolic blood pressure (DBP) (p < 0.001) was found in these patients. Finally, the group of higher AAC grade had higher corrected calcium (p = 0.004) and lower intact-parathyroid hormone (PTH) level (p = 0.037).

### Detail characteristics of AVF patency loss events

Table 2 reveals the detail characteristics of failing AVF. A total of 183 patency loss events of AVF occurred during the follow-up period, and all subjects were transferred for percutaneous intervention. Among these subjects before receiving intervention, 83 (45.4%) had inadequate AVF blood flow, 82 (44.8%) had occlusion, 10 (5.5%) had elevated venous pressure and 6 (3.3%) had difficult cannulation and limited cannulation site. The locations of stenosis were as follows: 125 (68.3%) arteriovenous anastomosis, 52 (28.4%) feeding artery, 88 (48.1%) draining vein and 115 (62.8%) juxta-anastomotic segment. Of these, 7mm (43.7%) of balloon angioplasty was the most common maximum diameter used in the procedure, follow by 6mm (30.6%) of balloon diameter. Pharmacologic thrombolysis was applied in 11 (6.0%) patients, where mechanical thrombolysis was performed in 9 (4.9%) patients. Finally, complications noted as venous rupture were experienced in 4 (2.2%) of the procedures.

### Factors associated with primary patency loss of arteriovenous fistula

**Figure 1:**
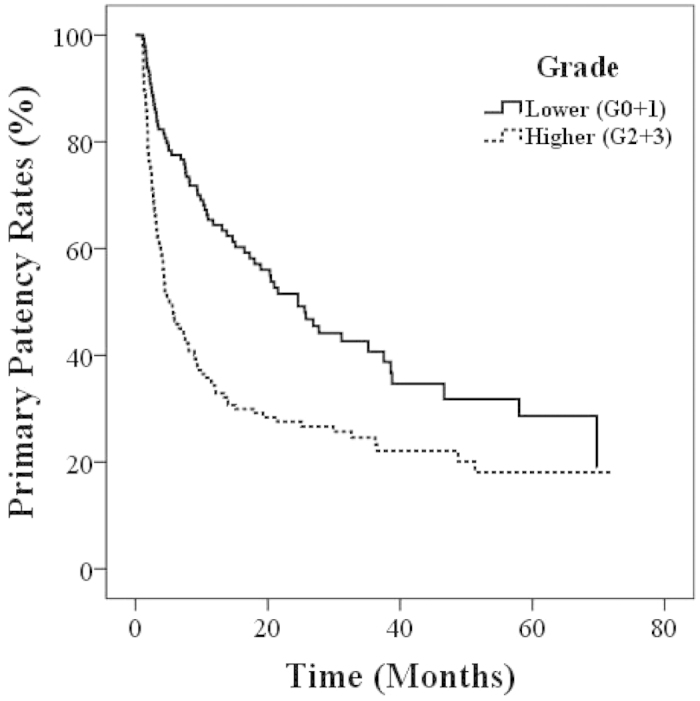
Kaplan–Meier curves for primary AVF patency in patients with lower grade (grades 0 + 1) and higher grade (grades 2 + 3) aortic arch calcifications. Log-rank *p* < 0.001.

Figure 1 shows the Kaplan–Meier survival curves for primary patency stratified by lower (grade 0 or 1) and higher AAC grades (grade 2 or 3). The patency rates at 6, 12, 24 and 36 months were 77.5%, 64.4%, 51.5% and 40.7% for lower grade and 46.5%, 34.3%, 27.6% and 24.6% for higher grade respectively. Kaplan–Meier analysis established that the incidence of patency loss events were significantly higher in patients with higher AAC grade compared to those with lower AAC grade (log-rank test; p < 0.001).

Table 3 shows the univariate Cox proportional hazard results of AVF primary patency loss for all relevant factors. After univariate analysis, grade 2 (1.66 (1.09–2.54); p = 0.018) and grade 3 (2.50 (1.59–3.93); p < 0.001) of AAC were associated with an increased risk of patency loss. Table 4 shows the adjusted hazard ratio of the risk factors for AVF patency loss by a multivariate Cox proportional regression model. In all patients with AVF, the presence of AAC grade 2 (1.80 (1.15–2.84); p = 0.011) and grade 3 (3.03 (1.88–4.91); p < 0.001), and higher level of intact-PTH (p = 0.047) were associated with AVF patency loss. However, in subgroup analysis, which included AVF created by a surgeon assisted with preoperative vascular mapping, only AAC grade 3 (2.41 (1.45–4.00); p = 0.001), and higher intact-parathyroid hormone (p = 0.025) level were correlated with AVF patency loss. Of note, the presence of comorbid conditions, and surgeon without assistance with vascular mapping were not associated significantly with primary patency loss of AVF.

## Discussion

In this observation study, we demonstrated a high AAC prevalence rate of 79.0% in our ESRD patients, and 53.9% of them presented with higher AAC grade (grades 2 to grade 3). In addition, we found that higher AAC grade and intact-PTH level predicted primary patency loss of AVF. To our knowledge, this is the first article to study the effect of AAC grade on primary patency rate of AVF.

In the present study, patients with higher AAC grade were older and had more cardiovascular disease or diabetes, indicating that these ESRD patients were more prone to multiple comorbid conditions. These results are in accordance with findings from previous studies^6^,^16^. In addition, higher calcium and lower intact-PTH levels were also associated with higher AAC grade, suggesting this condition is more likely to be correlated with vascular calcification, partly owing to the decreased calcium-buffering capacity of bone^17^. Of note, lower blood pressure was prevalent in the higher AAC grade group. One possible reason may be these high-risk populations had used more anti-hypertensive medication for cardio- or reno-protective effect due to multiple comorbid conditions.

Although the extent of vascular calcification can be quantified precisely with EBCT and MDCT, these expensive imaging modalities are not routinely used^14^,^15^. In contrast, the method by chest radiography for AVF risk stratification has the advantage of being less time-consuming and non-invasive. Besides, since it may also be applied for assessment of the cardiothoracic ratio, it is a relatively common and cost-effective screening tool that can be used in place of plain hand film in detecting vascular calcification among hemodialysis patients. Otherwise, the evaluation of AAC grading on chest radiography is a very simple tool, easy to use by the physician and can be done without the assistant of a radiologist^18^. In this context, chest radiography could be recommended as a standard screening method for vascular calcification in hemodialysis patients with an AVF.

The major finding of our study is that the presence of higher AAC grade is an important risk factor for primary patency loss of AVF. Recent studies confirmed that AAC was an independent determinant of cardiovascular outcome in both chronic kidney disease (CKD) and non-CKD populations^6^,^9^,^16^,^19^. Among these studies, several reports indicated that only patients with higher AAC grade were associated with increased cardiovascular mortality after adjustment for potential risk factors^9^,^16^,^19^. This result resembled the finding in our report which showed that AAC grade 1 was not significantly related to greater risk of access failure. It was proposed that trivial calcium deposition in aortic arch only might not be a noticeable vascular risk. Taken together, despite these many previous studies having shown a positive association of cardiovascular risk with AAC, there is relatively no report regarding the predictive value of AAC grade against vascular access patency.

There are several possible explanations for the links between AAC grade and loss of AVF patency. First, AAC could be a marker of systemic vascular calcification. It has been shown that patients with AAC had increased calcification of the coronary artery, heart valve or extra- and intracranial carotid artery^7^,^8^, and even had more frequent vascular access calcification^10^. Besides, the severity of AAC might reflect the degree of calcification in the whole aorta, and is highly correlated with the intima-media thickness of the carotid artery, and is indirectly associated with intima-media ratio of radial artery^20^,^21^. Moreover, a previous investigation confirmed that patients with higher AAC grade had impaired brachial flow-mediated dilation (FMD) or nitroglycerine-mediated dilation (NMD), and both these measurements are indicators of artery stiffness, probably owing to preexistence of microscopic calcification in the arterial media^19^. In fact, both increased radial artery intima-media thickness and preexisting vascular access calcification might predict patency loss of AVF in ESRD patients as established by prior reports^11^,^22^,^23^. Second, it is well known that both AAC and failed AVF share several common atherosclerotic risk factors such as older age, diabetes, cardiovascular disease and etc. To our knowledge, these risk factors as well as other undetermined variables could contribute to AVF dysfunction^3^,^8^,^19^,^24^. Third, the link between AAC and inflammation in ESRD patients makes the former a competent determinant of AVF dysfunction. Based on recent review, cumulative evidence emphasizes inflammation and oxidative stress signaling as key contributors to the pathogenesis of vascular mineral deposition^25^. Chronic inflammation accelerates vascular calcification, and in clinical practice, overexpression of inflammatory cytokines, such as interleukin (IL)1β, IL-6, C-reactive protein (CRP) and tumor necrosis factor (TNF) on a failed vascular access strengthens the inflammatory hypothesis of AVF failure^5^,^26^.

The potential mechanisms between vascular calcification and adverse AVF outcome have been well studied. Calcification can occur in both the intimal and medial layers of vasculature, but medial calcification is more prominent in ESRD patients^4^. In fact, intimal calcification is associated with the development of plaques and occlusive lesions, whereas medial calcification is a nonocclusive process which leads to increased vascular stiffness and reduced vascular compliance^27^,^28^. In our report, we were unable to distinguish these two calcified lesions by only using plain radiography, but both types of calcification are believed to be able to cause access dysfunction directly, including early thrombosis and non-maturation^11^,^29^.

In concordance with a previous study, higher intact-PTH level has also been recognized as a risk factor for AVF patency loss^30^. This result is well in agreement with a prior finding showing elevated intact-PTH level was significantly associated with an increased risk of atherosclerotic disease^31^. It has been proposed that hyperparathyroidism may participate in the pathogenesis of atherosclerotic lesion as well as AVF thrombosis through an increased calcium-phosphorus product^32^. Additionally, one could postulate that higher intact-PTH level, which was related to impaired endothelial function and increased aortic pulse pressure, may also help explain previously observed correlations of elevated intact-PTH with both atherosclerosis disease and failing AVF^33^.

Notably, our result demonstrated that there was no significant difference regarding the outcome of AVF among surgeons with or without assistance of preoperative vascular mapping. This can be explained by vascular mapping perhaps decreasing immediate failure rate of AVF, but probably not for primary patency and complication rates^34^. Besides, a systemic review also indicated that vascular mapping was not significantly better than clinical evaluation in terms of AVF primary patency^35^. However, there was much unequal distribution of vascular mapping assessment numbers among these surgeons in our study; hence, this result might be inconclusive. Besides, in patients assisted with preoperative vascular mapping, there was no more significant impact of AAC grade 2 on AVF patency. It was hypothesized that surgeon experience assisted with ultrasonography could easily and precisely screen and exclude high risk patients particularly those with calcified vessels^36^, and these populations who probably consist of high grade AAC might be transferred for AVG creation or permanent catheter placement. For this reason, a future large clinical trial is required to ascertain the role of ultrasonography in these patients with a vascular access.

There are several limitations of our study. First, the evaluation of AAC was performed using a simple chest radiography technique; hence, we might have missed early stages of calcification such as microcalcifications in healthy vessels. However, there was no effect on AAC grading since these early stages of calcification would be categorized as lower AAC grade. Second, it was a single center and observational study, and study subjects may not be generalized to all ESRD population. Third, given the retrospective nature of the study, certain potential factors like inflammatory markers, dynamic measurements and vascular calcification of AVF and time period of puncture after AVF implantation were unavailable in medical records and thus not included in the analysis.

In conclusion, our results show that higher AAC grade (grade 2 and grade 3) and higher intact-PTH level were associated with greater risk of primary patency loss of AVF. The grading of AAC by simple chest radiography represents a cost-effective and easy-to-perform method to identify patients at increased access failure risk. Thus, routine chest radiography as a screening or risk factor assessment tool may be considered in these ESRD populations for AVF creation.

## Methods

### Study design and patients

This was a retrospective cohort study performed in a single center, utilizing the medical records of all ESRD patients who had construction of AVF for chronic hemodialysis. Between January 2007 and June 2014, 286 patients who underwent surgery to create AVF were enrolled as study subjects. Most subjects were involved in a Pre-ESRD program (providing appropriate care to the ESRD population), and they were recruited consecutively from the outpatient department of nephrology. Eligibility included patients with age older than 20 years, had undergone first AVF surgery, and had an estimated glomerular filtration rate (eGFR) of <15 ml/min/1.73 m^2^ based on the modification of diet in renal disease (MDRD) study formula. Subjects were excluded from the study if they experienced AVF failure within 1 month or length of follow-up period less than 1 month after surgery (n = 22), or those who had no chest x-ray records (n = 5). All subjects were followed up from the creation of first AVF until transplantation, mortality, transferal to another center, or 31 December 2014. This study protocol was approved by the Institutional Review Board of Yuan’s General Hospital, and the methods were performed in accordance with the guidelines of human subject research developed by the Taiwan Ministry of Health and Welfare. Requirement for patient consent was waived due to the retrospective nature of the study and minimal participant risk.

### Demographic and clinical data

Data on demographic data, comorbidities, concomitant medications, body mass index (BMI), blood pressure, AVF characteristics and laboratory parameters were reviewed from medical records prior to surgery. The presence of CAD was diagnosed if they had a history of typical angina, old myocardial infarction, or they had undergone coronary artery bypass surgery or angioplasty. CVA was diagnosed by a history of cerebrovascular incidents such as cerebral bleeding or infarction. PAD was defined by prior revascularization, amputation for ischemia or gangrene, or an ankle-brachial pressure index of <0.9. Diabetes was defined if fasting plasma glucose levels >6.99 mmol/L, or glycated hemoglobin (HbA1c) > 6.5%, or if the patient was currently using hypoglycemic agents. Hypertension was defined if they had SBP ≥140 mm Hg, DBP ≥90mmHg or a filled prescription for an antihypertensive medication. Prior dialysis represented that patients had initial dialysis therapy before receiving AVF implantation. BMI was calculated dividing body weight (kg) by body height (m)^2^. Blood pressure was measured with using an automatic oscillometric monitor.

All AVFs were performed directly by three experienced surgeons (defined as A, B and C). Preoperative sonographic vascular mapping was only performed routinely by surgeon C (85% of study subjects), for the purpose to identify vessels suitable for access creation by setting minimum vascular diameters and ensuring vessel patency. The decision regarding type and location of the initial access creation was individually based on clinical finding and surgeons’ opinion. Normally, a forearm fistula was created preferentially, but upper arm fistulas or grafts were placed when the forearm vessels were inadequate.

All laboratory measurements were performed by our hospital laboratory using standardized and automated methods. Otherwise, calcium concentrations (mg/dL) were corrected for albumin concentrations (g/dL) using the following formula: corrected calcium = calcium + 0.8 × (4 − albumin). The eGFR was estimated using the MDRD Study formula as follows: 186 × [serum creatinine (mg/dL)]^−1.154^ × (age)^−0.203^ × (0.742 if female)^37^.

### Assessment of aortic arch calcification

The earliest available (closest to date of access placement), technically adequate chest X-ray was chosen for review. We recognized technical adequacy as a posterior-anterior view of chest X-ray which exhibiting a defined aortic knob. X-ray images were analyzed by two experienced physicians who were blinded to the patient’s condition. In all cases of disagreement between the physicians, consensus was eventually reached. The grade of AAC was assessed using a previously validated scoring system: grade 0 (no visible calcification), grade 1 (small spots of calcification or single thin calcification of the aortic knob), grade 2 (one or more areas of thick calcification, but ≤50% of the circular area of the aortic knob), and grade 3 (circular calcification with >50% of circular area of the aortic knob)^18^. Grades 0 to 1 and grades 2 to 3 were categorized as lower and higher AAC grade respectively.

### Study outcome and variables of failing AVF

The duration of primary patency was defined as the period from AVF placement to first access occlusion or any intervention for restoring blood flow^38^,^39^. As such, these conditions included inadequate AVF blood flow, occlusion, elevated venous pressure, difficult cannulation and limited cannulation site, and other complications leading to nonfunctional access. Of note, skillful physical examination has been recognized as a valuable tool in assessing failure of AVF^40^. Furthermore, the variables of failing AVF were noted as presentation, the presence of single or multiple segmental stenoses, location of stenosis, maximum diameter of balloon angioplasty, method of thrombolysis and complication. In terms of location of the stenosis, the AVF was categorized into 4 segments: the arteriovenous anastomosis, the feeding artery, the draining vein and the juxta-anastomotic segment of the fistula. For the thrombolysis method, it was divided into pharmacologic thrombolysis (urokinase/heparin use) and mechanical thrombolysis (aspiration thrombectomy).

### Statistical analysis

Quantitative variables were presented as mean ± standard deviation or median (interquartile range) if they were normally or non-normally distributed, while categorical variables were presented as number with percentages. The study groups stratified by aortic arch calcification were compared on baseline characteristics using Chi-square test or Fisher’s exact test for categorical variables, independent t-test for continuous variables and Mann-Whitney test for intact-PTH.

Survival curve for primary patency loss was determined with the Kaplan–Meier method, and the comparisons between lower and higher grades of AAC were done with the log-rank test. Initially, a univariate Cox proportional hazard analysis was done with variables considered relevant to patency loss of arteriovenous access. After that, multivariate Cox proportional hazards models (stepwise method) were used to determine factors associated with reduced access patency after adjusting for all potential variables. For the subgroup analysis that focused on the vascular mapping group, multivariate Cox proportional hazards models were also performed to determine factors associated with AVF patency loss. Test results were presented as hazard ratios (HR) with 95% confidence intervals (CI), and two-sided *p* < 0.05 was considered statistically significant. Statistical analysis was performed with SPSS software (SPSS Version 19; SPSS Inc., Chicago, IL, USA).

## Additional Information

**How to cite this article**: Yap, Y.-S. *et al.* Aortic Arch Calcification Predicts Patency Loss of Arteriovenous Fistula in End-Stage Renal Disease Patients. *Sci. Rep.* **6**, 24943; doi: 10.1038/srep24943 (2016).

## Figures and Tables

**Table 1:** Baseline characteristics and comparisons of patients with Grades 0 to 1 and Grades 2 to 3 of aortic arch calcification.

Characteristics	Total (n = 286 )	Aortic arch calcification		*P* value
		Lower grade, grades 0 to 1 (n = 132, 46.2%)	Higher grade, grades 2 to 3 (n = 154, 53.8%)	
Age, years	62.5 ± 13.1	56.9 ± 13.7	67.3 ± 10.4	<0.001*
Gender, n (%)				
Male	163 (57.0)	84 (63.6)	79 (51.3)	0.036*
Female	123 (43.0)	48 (36.4)	75 (48.7)	
Comorbid conditions, n (%)				
Coronary artery disease	91 (31.8)	26 (19.7)	65 (42.2)	<0.001*
Cerebrovascular disease	52 (18.2)	14 (10.6)	38 (24.7)	0.002*
Peripheral artery disease	29 (10.1)	11 (8.3)	18 (11.7)	0.349
Diabetes mellitus	214 (74.8)	89 (67.4)	125 (81.2)	0.008*
Hypertension	279 (97.6)	126 (95.5)	153 (99.4)	0.051
Prior dialysis	186 (65.5)	90 (68.7)	96 (62.7)	0.292
Limb of AVF, n (%)				
Left	206 (72.0)	95 (74.8)	111 (72.5)	0.670
Right	74 (25.9)	32 (25.2)	42 (27.5)	
Location of AVF, n(%)				
Forearm	244 (85.3)	109 (85.8)	135 (88.2)	0.549
Upper arm	36 (12.6)	18 (14.2)	18 (11.8)	
Surgeon				
A without vascular mapping	20 (7.0)	10 (7.6)	10 (6.5)	0.300
B without vascular mapping	23 (8.0)	14 (10.6)	9 (5.8)	
C with vascular mapping	243 (85.0)	108 (81.8)	135 (87.8)	
Body mass index, kg/m^2^	25.0 ± 5.2	25.1 ± 6.0	24.9 ± 4.5	0.792
Systolic blood pressure, mmHg	143 ± 22	146 ± 23	141 ± 21	0.046*
Diastolic blood pressure, mmHg	79 ± 13	82 ± 13	77 ± 11	<0.001*
Laboratory test				
MDRD-GFR, ml/min/1.73 m^2^	5.3 ± 1.7	5.3 ± 1.8	5.3 ± 1.7	0.831
Hemoglobin, g/L	88 ± 15	88 ± 14	88 ± 15	0.962
Uric acid, mmol/L	0.52 ± 0.14	0.51 ± 0.13	0.53 ± 0.15	0.172
Corrected calcium, mmol/L	2.14 ± 0.23	2.10 ± 0.25	2.18 ± 0.20	0.004*
Phosphorus, mmol/L	1.96 ± 0.64	2.01 ± 0.67	1.91 ± 0.61	0.164
Intact-PTH, pmol/L	20.3 (10.7–33.0)	25.2 (11.6–38.0)	18.4 (9.4–26.9)	0.037*
Serum albumin, g/L	34 ± 6	34 ± 6	34 ± 5	0.833
Total cholesterol, mmol/L	4.47 ± 1.39	4.56 ± 1.39	4.39 ± 1.39	0.305
Triglyceride, mmol/L	1.75 ± 1.20	1.76 ± 1.31	1.73 ± 1.11	0.845
Medications				
Anti-platelet agent	122 (42.7)	49 (37.1)	73 (47.4)	0.080
ACEI/ARB	205 (71.7)	92 (69.7)	113 (73.4)	0.491
Statin/fibrate	92 (32.2)	41 (31.1)	51 (33.1)	0.711

Abbreviations: AVF = Arteriovenous fistula; MDRD = Modification of diet in renal disease; GFR = Glomerular filtration rate; PTH = Parathyroid hormone; ACEI = Angiotensin converting enzyme inhibitor; ARB = Angiotensin receptor blocker. Data are presented as mean ± standard deviation or numbers (percentages), except for intact-PTH, which are presented as median (interquartile range). *p < 0.05.

**Table 2:** Detailed characteristics of AVF patency loss events (n = 183).

Characteristics	Value
Age, years	62.7 ± 12.6
Gender, n (%)	
Male	103 (56.3)
Female	80 (43.7)
Limb of AVF, n (%)	
Left	132 (72.1)
Right	49 (26.8)
Location of AVF, n (%)	
Forearm	163 (89.1)
Upper arm	18 (9.8)
Presentation of failing AVF, n (%)	
Inadequate blood flow	83 (45.4)
Occlusion	82 (44.8)
Elevated venous pressure,	10 (5.5)
Difficult cannulation/limited cannulation site	6 (3.3)
Character of stenosis, n (%)	
Single segmental	86 (47)
Multiple segmental	87 (47.5)
Location of stenosis, n (%)	
Arteriovenous anastomosis	125 (68.3)
Feeding artery	52 (28.4)
Draining vein	88 (48.1)
Juxta-anastomotic segment	115 (62.8)
Maximum diameter of balloon angioplasty, n (%)	
≦5 mm	21 (11.5)
6 mm	56 (30.6)
7 mm	80 (43.7)
≧8 mm	16 (8.7)
Method of thrombolysis, n (%)	
Pharmacologic thrombolysis	11 (6.0)
Mechanical thrombolysis	9 (4.9)
Complication, n (%)	
Venous rupture	4 (2.2%)

Abbreviations: AVF = Arteriovenous fistula.

**Table 3:** Univariate Cox proportional hazards analysis for primary patency loss of AVF.

Factors	Comparison	HR (95%C.I.)	P value
Age	Per 10 years increase	1.08 (0.97–1.21)	0.162
Sex	Male	1	
	Female	1.12 (0.83–1.50)	0.460
Comorbid conditions			
Coronary heart disease	No	1	
	Yes	1.09 (0.80–1.48)	0.577
Cerebrovascular disease	No	1	
	Yes	0.93 (0.63–1.37)	0.72
Peripheral artery disease	No	1	
	Yes	1.17 (0.73–1.86)	0.511
Diabetes mellitus	No	1	
Hypertension	Yes	1.32 (0.93–1.87)	0.125
	No	1	
	Yes	1.42 (0.53–3.82)	0.492
Prior dialysis	No	1	
	Yes	0.85 (0.63–1.14)	0.278
Limb of AVF	Left	1	
	Right	1.21 (0.87–1.68)	0.256
Location of AVF	Forearm	1	
	Upper arm	0.76 (0.47–1.24)	0.276
Surgeon	A without vascular mapping	1	
	B without vascular mapping	0.93 (0.54–1.61)	0.789
	C with vascular mapping	1.56 (0.78–3.12)	0.206
Body mass index	Per 1 kg/m^2^ increase	1.02 (0.99–1.04)	0.273
Systolic blood pressure	Per 10 mmHg increase	0.94 (0.88–1.01)	0.105
Diastolic blood pressure	Per 10 mmHg increase	0.89 (0.78–1.01)	0.066
Laboratory test			
MDRD-GFR	Per 1 ml/min/1.73 m^2^ decrease	0.97 (0.89–1.06)	0.518
Hemoglobin	Per 10 g/L decrease	1.06 (0.96–1.18)	0.252
Uric acid	Per 0.059 mmol/L increase	1.01 (0.95–1.08)	0.689
Corrected calcium	Per 0.25 mmol/L increase	0.98 (0.84–1.15)	0.793
Phosphorus	Per 0.323 mmol/L increase	0.99 (0.92–1.07)	0.788
Intact-PTH	Per 10.6 pmol/L increase	1.04 (0.97–1.11)	0.246
Serum albumin	Per 10 g/L decrease	1.21 (0.94–1.55)	0.136
Total cholesterol	Per 0.26 mmol/L increase	0.99 (0.96–1.02)	0.534
Triglyceride	Per 0.11 mmol/L increase	1.00 (0.99–1.01)	0.770
Medication			
Anti-platelet agent	No	1	
	Yes	1.09 (0.82–1.46)	0.553
ACEI/ARB	No	1	
	Yes	0.76 (0.55–1.03)	0.079
Statin/fibrate	No	1	
	Yes	0.91 (0.66–1.24)	0.533
Aortic arch calcification	Grade 0	1	
	Grade 1	0.99 (0.62–1.59)	0.979
	Grade 2	1.66 (1.09–2.54)	0.018*
	Grade 3	2.50 (1.59–3.93)	< 0.001*

Abbreviations: AVF = Arteriovenous fistula; HR = Hazard ratio; C.I. = Confidence interval; MDRD = Modification of diet in renal disease; GFR = Glomerular filtration rate; PTH = Parathyroid hormone; ACEI = Angiotensin converting enzyme inhibitor; ARB = Angiotensin receptor blocker. *p < 0.05.

**Table 4:** Multivariate Cox proportional hazards analysis for primary patency loss of AVF of all and subgroup patients.

Factors	Comparison	HR (95%C.I.)	P value
All patients (n = 286)			
Intact-PTH	Per 10.6 pmol/L increase	1.06 (1.00–1.13)	0.047
Aortic arch calcification	Grade 0	1	
	Grade 1	1.06 (0.65–1.74)	0.815
	Grade 2	1.80 (1.15–2.84)	0.011
	Grade 3	3.03 (1.88–4.91)	<0.001
Subgroup patients (n = 243)			
Intact-PTH	Per 10.6 pmol/L increase	1.08 (1.01–1.15)	0.025
Aortic arch calcification	Grade 0	1	
	Grade 1	0.82 (0.48–1.40)	0.464
	Grade 2	1.39 (0.87–2.24)	0.173
	Grade 3	2.41 (1.45–4.00)	0.001

Abbreviations: HR = Hazard ratio; C.I. = Confidence interval; PTH = Parathyroid hormone. Subgroup patients were defined as patients in whom AVFs were created by a surgeon assisted with preoperative vascular mapping. All variables were included into multivariate analysis, and variables were dropped from the table if significant p > 0.05.
